# Administration of antioxidants in chronic kidney disease

**Published:** 2015-01-01

**Authors:** Mohamad Reza Tamadon, Mehrdad Zahmatkesh, Seyed Seifollah Beladi Mousavi

**Affiliations:** ^1^Department of Internal Medicine, Faculty of Medicine, Semnan University of Medical Sciences, Semnan, Iran; ^2^Kowsar Hospital, Semnan University of Medical Sciences, Semnan, Iran; ^3^Chronic Renal Failure Research Center, Ahvaz Jundishapur University of Medical Sciences, Ahvaz, Iran

**Keywords:** Chronic kidney disease, Antioxidants, Hemodialysis

**Implication for health policy/practice/research/medical education:**

Majority of studies indicated that, antioxidants are helpful in hemodialysis patients, reducing the risk of cardiovascular events and increasing the quality of dialysis.

Chronic kidney disease has been a health problem in the past, however, today has become a global health threat. The number of chronic renal failure patients is increasing and more than one million people in the end-stage of chronic renal failure are dying annually (1). These patients experience various problems and creating a change in their life is very useful. Fatigue is a common problem in dialysis patients. Fatigue is the most important complication of vitamin C deficiency. Vitamin C is a well-known antioxidant (2).

Hemodialysis process decreased the necessary antioxidants and chronic renal failure associated with stress oxidative (3). Excessive production of free radicals is a state that called stress oxidative which is one of the reasons for vascular lesions (4). Free radicals affect on carbohydrates, protein, fat and DNA (5,6). Free radicals cause lipid peroxidation and degradation of molecules and cellular structures (endothelial cells and red blood cells) (7). Some studies have indicated to the increasing of free radicals caused by dialysis (8-10). Small proteins such as immunoglobulin G and complements attached to the dialyzer membrane and activate granulocytes which resulting in production of free radicals (11,12).

One of the main causes of death in chronic dialysis patients is cardiovascular events. Increasing of peroxidation products and also antioxidant depletion are effective actors of atherosclerosis in patients who undergoing hemodialysis (13).

Chronic kidney disease associated with high incidence of cardiovascular disease which is common cause of mortality and also imposes high costs (5,6). Chronic renal insufficiency even if you eliminate the initial cause progress to end-stage renal disease, because the initial injury eventually leads to scarring and loss of renal nephrons and resulting in end-stage kidney disease (7-14).

Various investigations mentioned the positive effects of antioxidants in chronic diseases, cardiovascular diseases, hypertension and kidney disease, although some studies have been reported no beneficial effect on reducing mortality and cardiovascular disease (15-21).

Antioxidants are in foods and some studies mentioned to their beneficial role in chronic kidney disease or hypertension (22,23).

This article, reviews some articles regarding the role of antioxidants in hemodialysis patients.

We searched scientific sources and article index databases including PubMed and Scopus by key words including antioxidants, antioxidant therapy in hemodialysis patients, hemodialysis and antioxidants. From the existing articles we reviewed 48 articles.

In study by Santana-Santos *et al.* (24), administration of N-acetylcysteine was effective in reducing of acute kidney injury in patients with kidney disease who underwent CABG surgery and mentioned that prevented from oxidative stress.

However, other study implied that it had not any effect in acute renal injury and chronic kidney disease (25).

Tbahriti *et al*. (26), in their study detected the antioxidant enzyme activities change under the influence of renal dysfunction and dialysis.

In another study prescription of an antioxidant, alpha lipoic acid has been useful for diabetic and dialysis patients, while, the most common cause of reaching to the end-stage renal disease in the most communities is diabetes (27).

However, various studies have been reported conflicting results. In a study, antioxidants have been effective in patients with non-dialysis kidney diseases but were not effective in dialysis patients (28-30). Additionally, the impact of anti-oxidants to reduce mortality resulting from cardiovascular diseases has been of much interest (31-33).

Some studies reported that cytokines level is higher in hemodialysis patients and have important pathological role in oxidative stress, progression of diabetes complications and increasing the oxidative stress intensity after hemodialysis and emphased to antioxidants benefits in diabetic and end-stage renal disease patients who are under dialysis (34-36).

It is possible that, reducing the activity of antioxidant enzyme in red blood cells and increasing of lipid peroxidation in hemodialysis patients play a role in progression of cardiovascular disease and antioxidants are effective to reduce cardiovascular events (37-39).

Moreover, some studies point out that the use of dialyzer with antioxidant membrane, taking vitamin D and iron supplements and prescribed antioxidants such as vitamin E and C increase the quality of dialysis and reduce the incidence of oxidative stress and some complications (40-45). Likewise, the effect of low doses of vitamin C on the inflammatory process and reducing of fatigue in hemodialysis patients was emphasized in recent studies (46-48).

According to these findings more studies are necessary to exactly find the effects of antioxidants in hemodialysis patients and reducing complications from dialysis and kidney disease. We recommend to assess their effects and determining a comprehensive therapeutic protocol for antioxidants therapy in hemodialysis patients.

## Conclusion

Majority of studies indicated that, antioxidants are helpful in hemodialysis patients, reducing the risk of cardiovascular events and increasing the quality of dialysis.

## Authors’ contributions

All authors contributed to the manuscript equally.

## Conflict of interests

The authors declared no competing interests.

## Ethical considerations

Ethical issues (including plagiarism, misconduct, data fabrication, falsification, double publication or submission, redundancy) have been completely observed by the authors.

## Funding/Support

None.
